# Non-Destructive Evaluation Device for Monitoring Fluid Viscosity

**DOI:** 10.3390/s20061657

**Published:** 2020-03-17

**Authors:** Ahmed Abdulkareem, Ugur Erturun, Karla Mossi

**Affiliations:** 1 Department of Mechanical and Nuclear Engineering, Virginia Commonwealth University, Richmond, VA 23284, USA; abdulkareeam@mymail.vcu.edu; 2 Department of Electrical and Computer Engineering, John Hopkins University, Baltimore, MD 21218, USA; uerturu1@jhu.edu

**Keywords:** viscosity, piezoelectricity, wave propagation, non-destructive evaluation

## Abstract

There is an increasing need for non-destructive, low-cost devices for real-time fluid viscosity monitoring. Therefore, in this study, a method based on structural health monitoring is adapted for monitoring fluid properties. A device is built such that an inexpensive and disposable viscosity probe be possible. The design incorporates a sensor/actuator pair using a piezoelectric material layered with copper/brass and capable of monitoring viscosity changes in low volume liquids (e.g., vacutainer vial). Experiments performed with the new device show a definite pattern of wave propagation in viscous solutions. A numerical model is built to investigate the wave propagation in the fluid. For experimental measurements, the sensor part of the device detects the generated pressure wave in fluid (e.g., air, water, glycerin) by the actuator part. The phase shift between the actuator and the sensor signals is then recorded and plotted for different concentrations of glycerin and water at room temperature. The results of this study show a direct correlation between the phase shift and varying viscosity in the ultrasonic frequency range from 6 to 9 MHz. The numerical simulation, performed utilizing acoustic modal and harmonic response analysis, results also demonstrate the same trend as the experimental results: a phase shift increases with the viscosity of the fluid.

## 1. Introduction

Real-time inline viscosity monitoring of fluids is vital in many different industries ranging from the oil industry to healthcare applications [1,2,3,4,5]. For instance, in the oil and petroleum industry, viscosity measurements play a vital role in determining the oil quality that can affect the pipeline [6]. In the healthcare industry, monitoring blood viscosity is crucial for the treatment of some diseases, such as vascular-related diseases [7]. Standard laboratory viscometers, such as capillary tube and rotating ones, need long measurement times and a large volume sample [8]. Besides, rotating viscometers can be costly and require specialized training [1,8,9], and they are unable to measure viscosity in real-time continuously [1,3]. Thus, there is an increasing need for accurate, real-time, and low-cost viscosity measurement devices. Hence, a wide variety of devices have been developed over the years to establish high sensitivity, small size, easy of manufacture and use, real-time measurement, and low-cost [7,10,11,12]. Modern viscosity measurement devices can be categorized based on their operational principle of monitoring parameters (a) displacement, (b) vibrations, and (c) wave propagation in fluids of different viscosities [4,13,14]. 

A typical example of the first device category, a displacement approach, is based on correlating the viscosity of a fluid to the displacement of a ferromagnetic piston between two electromagnetic coils [15]. A recent method uses a fiber-optic sensor to measure the amplitude and phase responses to vibration without electromagnetic interference [11]. Zhang et al. [16] demonstrate a system that utilizes a polyvinylidene fluoride (PVDF) cantilever probe for actuation and a laser displacement sensor for detection. Though these devices are designed for specific applications, they usually work within a constrained low range of viscosity values and require specialized equipment such as optic fibers and lasers [17]. 

For the second category b, vibrations based, a wide variety of devices are available. One typical example consists of a piezoelectric cantilever sensor, which correlates the resonance frequency shift and fluid viscosity [18,19]. Similarly, measuring the resonance frequency of a vibrating beam by applying a Lorentz force generated by an Alternating Current (AC) in a permanent magnetic field is another mechanism that has been explored [20,21]. Lu et al. [22] propose a piezoelectric-excited membrane device using the PVDF membrane’s resonant frequency and *Q* factor responses to determine viscosity. Thickness shear mode resonators are other examples which use piezoelectric patches with electrodes on both sides [2,7]. The resonator is excited when an AC voltage is applied to the piezoelectric leads, thus changing the vibration characteristics for the resonator. The peak frequency of the resonator is affected by either the fluid medium [2,23] or a microcantilever structure by exerting an external force at the free end of the beam [3]. 

Similarly, quartz thickness-shear mode sensors are adapted to measure viscosity [7]. This sensor, in particular, monitors the process of platelet activation, which leads to thrombus formation (clotting). By following the sensors admittance frequency shifts concerning different concentrations of platelet activation, coagulation can be detected [7]. Purohit et al. [24] report a radial mode piezo-resonator disc to improve the ultrasonic determination of viscosity in liquids by correlating resonant frequency changes to viscosity. Another type worth mentioning is a capacitive micromachined transducer [12,25], which utilizes a non-destructive ultrasonic pulse-echo system using piezoelectric materials as membrane sensors and actuators. The changes to the waveform received due to mass changes correlate to viscosity. With the development of microelectromechanical systems (MEMS) technology, viscosity, and density measurements of different fluids are possible [5,10,26,27,28,29,30]. However, issues, including inaccurate estimation, low range of viscosities, lack of analytical models, and complex calibration procedures, remain a barrier with these types of devices [10,26,27].

The third type of devices measure the propagation time of wave in a fluid and correlate the result to the fluid viscosity. For instance, a piezoelectric sensor sends a signal to a liquid medium, and a reflector sends the signal back to the device [31]. The reflected wave impedance is then measured and correlated to viscosity [1,9]. This method requires a reflector and a large container and sample volume. This method has been widely used in structural health monitoring of solids or structures for years [31,32,33].

The work presented here proposes a new device that utilizes a method based on structural health monitoring and type c devices. In structural health monitoring, piezoelectric materials (such as lead zirconate titanate, PZT) are often used to sense and send waves to diagnose the mechanical failure of a structure, such as a crack or a loose fastener [32,33]. First, piezoelectric patches are attached to a structure, then using wave propagation, changes in the signal serve to identify structural changes by measuring impedance, current, etc. [33]. This method can be beneficial for the industrial sector as it does not need high-power devices or other sophisticated systems [32,33,34]. The drawbacks of these methods in structural health monitoring are scalability, and data processing can be quite complicated as the structures get large, more sensors and actuators are needed. These disadvantages, however, can be an advantage with viscous flows in small spaces.

In particular, this study aims to monitor fluid viscosity using a method similar to the one used in the non-destructive evaluation of structures. With the proposed approach, changes in the waveform of the signal received by a piezoelectric sensor partially submerged in different fluids can be correlated to viscosity. In this particular case, different viscosities are accomplished by mixing deionized (DI) water with glycerin. The goal is to demonstrate both numerically and experimentally that these techniques can be used to monitor bulk fluid properties such as viscosity, using ultrasound wave propagation in small volumes of fluid. This characteristic can be quite an advantage in specific applications, such as in the healthcare industry. An overview of the available methods to monitor viscosity, including the proposed method is shown in Table 1. 

## 2. Design and Fabrication

### 2.1. Design

**Figure 1:**
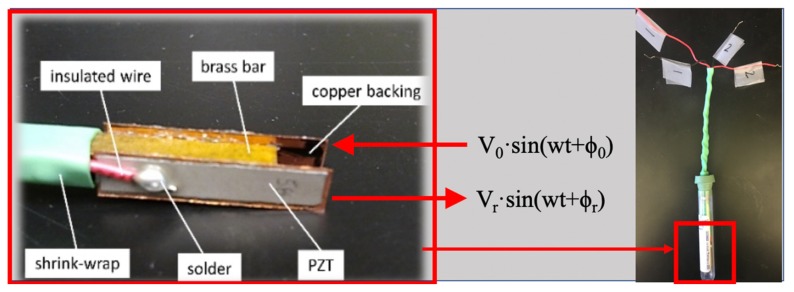
Probe construction and operation.

The design presented in this work consists of a piezoelectric sensor and actuator device pair based on a tuning fork principle using an actuator sensor pair. The device design constraints are size (small footprint, portability) and marketability (ease of manufacture, low cost, and expendable). A commercially available soft PZT (type 5A) was chosen for the sensor and actuator pair to satisfy marketability requirements. The PZT is bonded to a hollow brass tube, which is used to accommodate wiring on two opposite sides allowing the device to share a ground connection. The piezoelectric material that serves as an actuator vibrates as a function of the applied voltage signal, *V_o_ sin(wt+ϕ_o_)* where *V_o_* is the applied voltage at a particular frequency (*f = w/2π*), and an initial phase angle *ϕ_o_*= 0. The actuator produces sound pressure waves that travel approximately 5 mm through the fluid medium to the sensor that then records a waveform with an amplitude, *V_r_* or received voltage, and a phase angle *ϕ_r_*, *V_R_ sin(wt+ϕ_r_).* The proposed method is illustrated in Figure 1. 

### 2.2. Fabrication

#### 2.2.1. Materials

After considering several prototypes, the device built consists of layered materials bonded with a conventional adhesive at room temperature. The layers help to ensure structural integrity and ruggedness. These consisted of (a) two rectangular plates of PZT polarized with Nickel electrodes (23 × 5 mm with thickness of 1 mm by Morgan Matroc Ceramics); (b) two copper layers (25 × 6 mm with thickness of 1 mm); and (c) a rectangular hollow bar with a square cross-section made of brass (70 × 5 mm with thickness of 5 mm). The layers can be shown in Figure 1.

#### 2.2.2. Assembly

**Figure 2:**
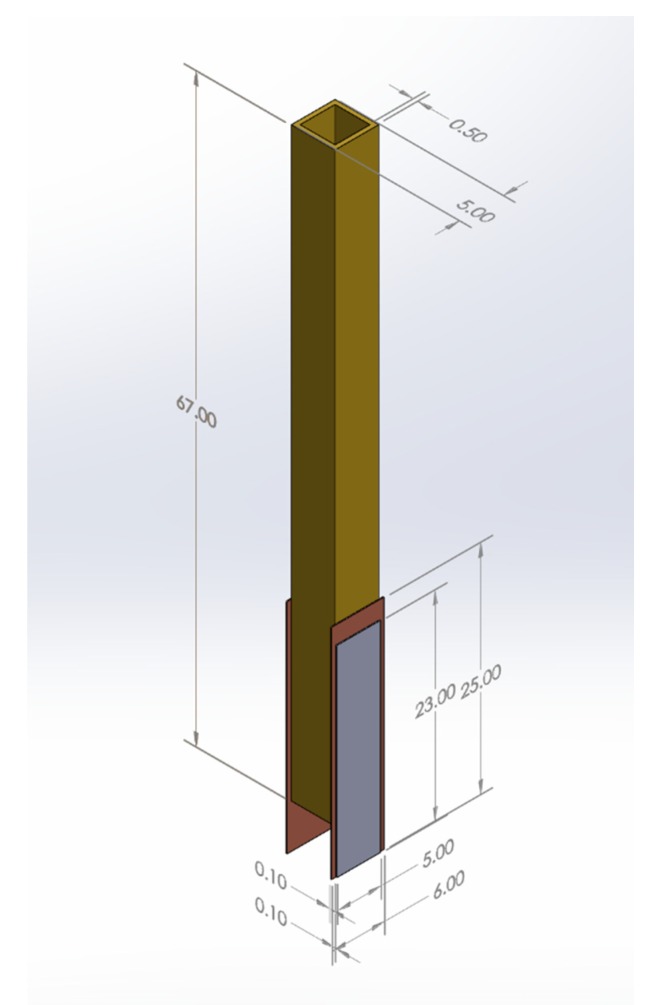
Final prototype dimensions in mm.

The assembly consists of a square hollow brass bar coated with polyethylene to provide electrical insulation. Then, two thin layers of Copper (0.1 cm) and PZT (0.1 cm) are placed with an adhesive to two of the outside opposing faces of the bar. Wires are soldered to the PZT surfaces for a positive connection, and the copper layer becomes ground. Heat shrink-wrap is used to secure the cables in place and minimize the footprint of the device. The final prototype fits easily inside a standard vacutainer (Lithium Heparin 56USP with a diameter of 13 mm, a capacity of 3 mL, and tube length of 75 mm). The final fabricated device and all dimensions are shown in Figure 2. 

## 3. Experimental Setup

Once the prototype is complete, a method that provides a consistent volume ratio of the several mixtures of glycerin and water is required. For experimental testing, the volume was kept constant at approximately 0.75 mm^3^ measured using a syringe. In this manner, only the tip of the probe is submerged in the liquid.

**Figure 3:**
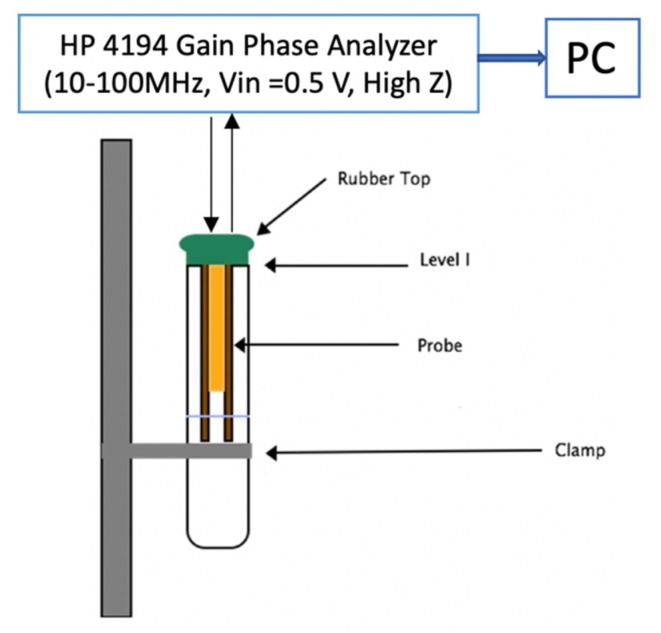
Experimental setup.

A LabView^®^ program records and controls a Hewlett Packard 4194A Impedance/Gain-Phase Analyser capable of measuring capacitance, impedance, gain, and phase angles. The analyzer scans frequencies and supply a sinusoidal wave to the actuator and monitor the signal received by the sensor. The applied signal is a sinusoidal wave with an amplitude of 0.5Volts at a range of frequencies between 100 Hz and 40 MHz. The measured gain and phase are the ratio of the amplitudes and the phase difference between the two signals. All measurements were performed in a custom-made Faraday cage to avoid interference in the high-frequency ranges. Figure 3 illustrates the experimental setup.

In addition to the Gain-Phase measurements, the impedance of the device in different liquids is also monitored. The range of frequencies is smaller since piezoelectric devices consume more current at the higher frequencies, and the impedance is not measured accurately at higher values. The following steps summarize procedures of a typical set of experiments: Measuring (1) the impedance of the actuator or sensor in water and glycerin; and; (2) the gain-phase with two different concentrations and mixtures of distilled water (DI) and glycerin. Once the experimental data is collected, it is analyzed using the software Sigma Plot. 

## 4. Modeling

**Figure 4:**
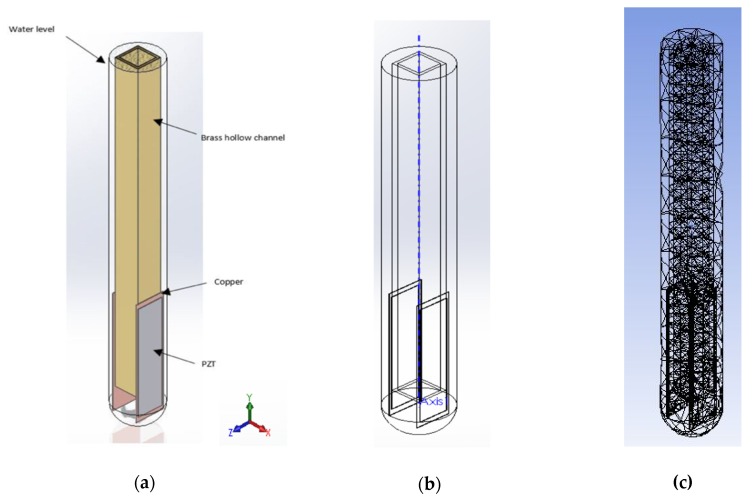
Model built in ANSYS: (**a**) schematic of the built model and fluid level; (**b**) the layers of the model; and (**c**) the mesh configuration.

A computer-generated model of the piezoelectric viscosity probe is built with dimensions of 5 × 5 × 70 mm, two copper sheets with dimensions of 6 × 0.1 × 25 mm, and two pieces of PZT type 5A with dimensions of 5 × 0.1 × 23 mm. Figure 4a and b show a diagram of the device assembly created using SolidWorks^®^.

Mechanical properties for all materials are listed in Table 2. All materials utilized are assumed to be isotropic except for the PZT material. The mesh of the probe and the surrounded fluid are assigned automatically using mesh element Solid 186 for the probe and Solid 187 for the surroundings. Mesh element Solid 186 has a quadratic displacement behavior, and each node contains three orthogonal translational degrees of freedom, which ensures the mesh is accurate and complete. For the fluid surrounding the probe, Fluid 220, and Fluid 221 is utilized. These last two elements have four degrees of freedom: the pressure and the three translational degrees of freedom, as shown in Figure 4c. Boundary conditions restrain the top of the probe to be consistent with the experiments to *u = u_x_ = u_y_ = u_z_ = 0*.

A three-dimensional finite element model was created using multi-physics software (ANSYS^®^ relase19R2). Using this software, eigenvalue frequencies (natural frequencies) for the piezoelectric viscosity probe can be calculated. However, if the probe is immersed in a fluid can have a significant effect on the calculation of natural frequencies and require additional tools [36]. A Fluid Solid Interaction module (FSI) that can couple the effects of the fluid and calculate the interactions is necessary. In this case, a harmonic acoustic analysis was performed for the probe and its surrounding fluid using a two-way Fluid Solid Interaction (FSI) method. This method is based on performing of a harmonic analysis for the actuator under the excitation voltage and then transferred effect of the acoustic wave due the excitation voltage to the sensor through the fluid medium using harmonic acoustic analysis. Then, an additional acoustic analysis is performed on the sensor to calculate the effect of the sound wave propagating in the fluid. In this case, the fluid is stationary and the fluid velocity is small and there is no need to solve for all the fluid dynamic parameters. Those effects are assumed negligible. 

In particular, the Fluid Solid Interaction (FSI) method is based on the solution of the equation of motion for the structure coupled with the Helmoholtz’s equation and the Navier–Stokes equation to include the fluid–solid interaction for the probe and the surrounded fluid. The complete finite element in matrix form for the fluid–solid interaction (FSI) is mathematically expressed as [37]:

(1) $$\left[\begin{array}{cc} \left[M_{s}\right] & \left[0\right]\\ \left[M_{\mathrm{fs}}\right] & \left[M_{f}\right] \end{array}\right]\left\{\begin{array}{c} \left\{\ddot{u}\right\}\\ \left\{\ddot{p}\right\} \end{array}\right\}+\left[\begin{array}{cc} \left[C_{s}\right] & \left[0\right]\\ \left[0\right] & \left[C_{f}\right] \end{array}\right]\left\{\begin{array}{c} \left\{\dot{u}\right\}\\ \left\{\dot{p}\right\} \end{array}\right\}+\left[\begin{array}{cc} \left[K_{s}\right] & \left[K_{fs}\right]\\ \left[0\right] & \left[M_{f}\right] \end{array}\right]\left\{\begin{array}{c} \left\{u\right\}\\ \left\{p\right\} \end{array}\right\}=\left\{\begin{array}{c} \left\{F_{s}\right\}\\ \left\{0\right\} \end{array}\right\},$$

where, [M_s_], [M_f_], [C_s_], [C_f_], [K_s_], [M_fs_], and [K_fs_] are the structural mass, the fluid mass, the structural damping, the fluid damping, the structural stiffness, the equivalent coupling “mass”, and the equivalent coupling “stiffness” matrices, respectively. In the same equation, {F_s_}, {u}, and {p} are the applied load vector, the nodal displacement, and the acoustic pressure, respectively. 

The piezoelectric material properties [38] for the PZT- 5A are defined as:

(2) $$\left[\frac{\varepsilon }{\varepsilon_{0}}\right]=\left[\begin{array}{c} 919\\ 0\\ 0 \end{array}\begin{array}{c} 0\\ 919.1\\ 0 \end{array}\begin{array}{c} 0\\ 0\\ 826.6 \end{array}\right],$$

(3) $$\left[e\right]=\left[\begin{array}{c} 0\\ 0\\ 0\\ 12.2947\\ 0\\ 0 \end{array}\begin{array}{c} -5.35116\\ 15.7835\\ -5.35116\\ 0\\ 0\\ 0 \end{array}\begin{array}{c} 0\\ 0\\ 0\\ 0\\ 12.2947\\ 0 \end{array}\right]C/m^{2},$$

(4) $$\left[C\right]=\left(\begin{array}{cccccc} 12.0346 & 7.5191 & 7.50901 & 0 & 0 & 0\\ 7.5191 & 12.0346 & 7.50901 & 0 & 0 & 0\\ 7.50901 & 7.50901 & 11.0867 & 0 & 0 & 0\\ 0 & 0 & 0 & 2.10526 & 0 & 0\\ 0 & 0 & 0 & 0 & 2.10526 & 0\\ 0 & 0 & 0 & 0 & 0 & 2.25734 \end{array}\right)\times 10^{10}\;\mathrm{Pa},$$

where [ε/ε_0_] is the relative permittivity, [*e*] is the coupling (in C/m^2^), and [C] is the elasticity (in Pa) matrices, respectively. In this manner, the model predicts the phase shift for the sensor immersed in a viscous flow under the same excitation voltage and range of frequency that had been used in the experimental work.

## 5. Results

The probe’s actuator is excited by applying a sinusoidal signal with 0.5 V (peak–peak voltage) in a range of frequencies from 100 Hz to 30 MHz. The probe’s sensor detects the generated wave in the fluid. The magnitude and the phase shift between the wave produced and the received wave is recorded. 

### 5.1. Experimental Phase Shift Measurements

**Figure 5:**
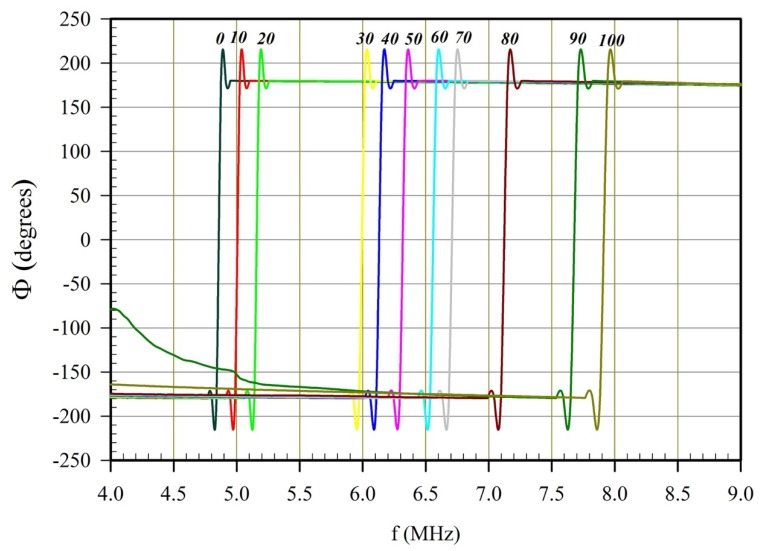
Peak phase shift vs. frequency as detected by the Gain-Phase Analyzer at different Glycerin concentrations (0 to 100%).

The measured and recorded phase shifts of the frequency in glycerin and DI-water (Deionized water) mixture of various concentrations are plotted for the probe, as shown in Figure 5. The phase shift is defined as the difference between the phase angle of the send signal and the received one. The frequency of the applied signal was scanned for the entire range the Gain-Phase analyzer is capable of performing (100Hz to 40Mhz). The largest peak phase sfhist was observed for all solutions to be in the higher frequency range. By taking the peak phase shift for each concentration, a pattern with an increasing percentage of the glycerin concentration (from 0 to 100%) is clear. In particular, note that at 100% DI water, the frequency shift can be detected at approximately 6.5 MHz. In contrast, a 100% glycerin solution showed the phase shift at a higher value, about 8.5 MHz. For a given range of concentration, this translates to a change in viscosity from 0.5 to 1600 cP. This pattern is observed for all different levels tested. Besides, a peak frequency at each concentration is measured, and that peak indicated a point where the acoustic pressure wave was maximum when traveling from the actuator to the sensor, under applied excitation voltage.

Since the probe is a prototype, there is room for some variability. Thus, quality assurance tests are crucial in determining prototype integrity. One way to monitor this integrity is periodically monitoring capacitance and impedance values since changes on the measured values may indicate delamination, cracks, or deficiencies in the probe. Phase shifts for each glycerin level are compared to known respective viscosity values, in the form of a calibration curve. Known glycerin viscosities regarding both temperature and solution percentage can be obtained from available literature [39]. A detailed error and p-values of the regression, as well as the values of the coefficient, are shown in Table 3. A statistical analysis of the regression is performed, and the *R^2^* of 0.99, indicating that the data fits a profile of the form is defined as follows.

(5) $$\nu =ae^{-\frac{1}{2}\cdot {\left(\frac{f_{R}-f_{0}}{b}\right)}^{2}},$$

where ν is the viscosity in cP, *f*_R_ is the resonance frequency (MHz), *f*_0_, and *a*, and *b* are constants that could be related to damping and sound propagation. Though the *R^2^* is high the parameters may not be quite accurate (coefficients have a high standard error and high *p*-values). This type of equation is usually used for a Gaussian wave equation and maybe significant in calibrating and predicting viscosity values. The large standard errors indicate multi-cocolinearity which indicates more refinement of this equation is needed and more data is required for a more definite model. The purpose of Equation (5) is to illustrate the trends observed in the experiments.

**Figure 6:**
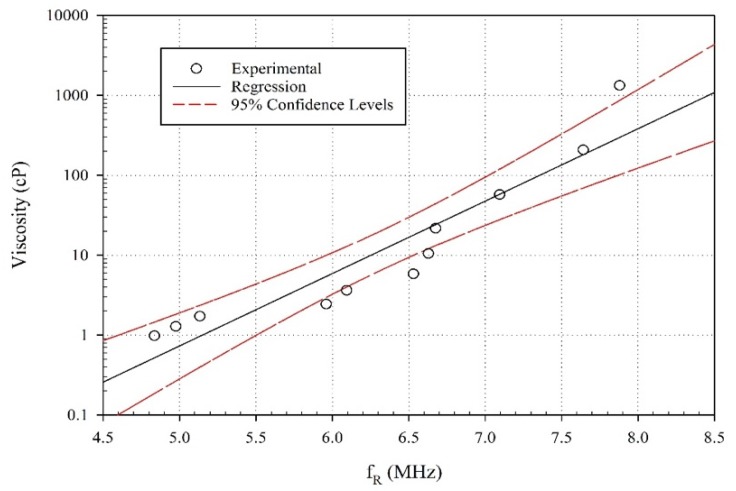
Frequency vs. viscosity regression.

The results of the regression and confidence intervals are shown in Figure 6 that illustrates the viscosity variation of different glycerin/DI water concentration levels with the frequency shifts. It can be seen from the graph that the frequency shift increases with increases of viscosity (increases in glycerin concentration).

**Figure 7:**
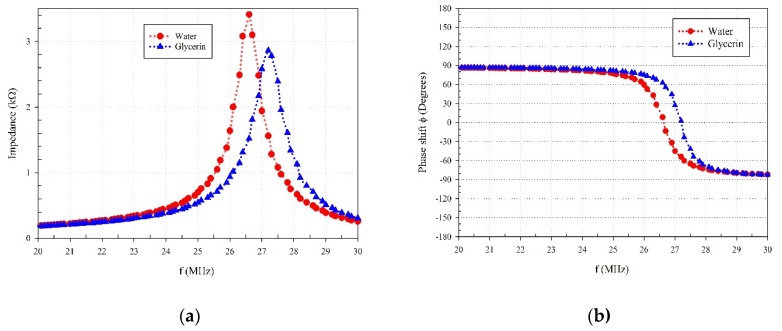
Measured (**a**) impedance of the probe in a fluid, and (**b**) the phase shift in a fluid.

The range of frequencies where significant changes in phase shifts and amplitude can be observed is the ultrasound range indicating the presence of acoustic pressure waves. The detailed work from Antlinger et al. [1] demonstrates that the wave propagation characteristics of a PZT device are affected by the fluid-solid interaction of the PZT. Furthermore, their work documents the electrical impedance of PZT resonators in a variety of fluids with different viscosities showing that the impedance amplitude diminishes when the liquid is more viscous [1]. They show that the resonance frequencies for a sensor surrounded by the fluid, under an excitation voltage, is affected by the electrical impedance which is a function of fluid parameters (frequency, speed of sound, viscosity, and damping) [1]. To that end, the impedance of the sensor/actuator pair used in the designed probe is measured, and the results can be seen in Figure 7. An impedance analyzer in the vacutainer measures the acoustic impedance of the PZT. There are differences between magnitudes and frequency peaks when submerged in different fluids. This behavior, as suggested in the work of Antlinger et al. [1], is the result of the electrical impedance of a piezoelectric actuator, which is the mechanism that creates an acoustic wave. However, the observed differences are too small to make inferences about the viscosity of the fluid medium. It does, however, provide an indication that the acoustic impedance is maximized at the MHz frequency.

### 5.2. Numerical Simulation Results 

The modal acoustic analysis with ANSYS was used to calculate the mode shapes of vibration for the probe and the surrounding fluid. In this manner, the natural resonant and vibration modes can be ruled out as a factor in the observed viscosity changes with frequency. The natural frequencies for the probe immersed into a water and glycerin solution are listed in Table 4. 

From Table 4, it can be seen that the order of magnitude for the resonance frequencies, based on the modal acoustic analysis for the probe, ranges from 683.86 to 2358 Hz for air, 500 to 1080 Hz for water, and 468 to 625 Hz for glycerin. Table 4 shows that the natural frequency in the air is higher than any of the natural frequencies when the probe is immersed in a fluid, possibly due to the dampening effect the fluid exerts on the probe. 

**Figure 8:**
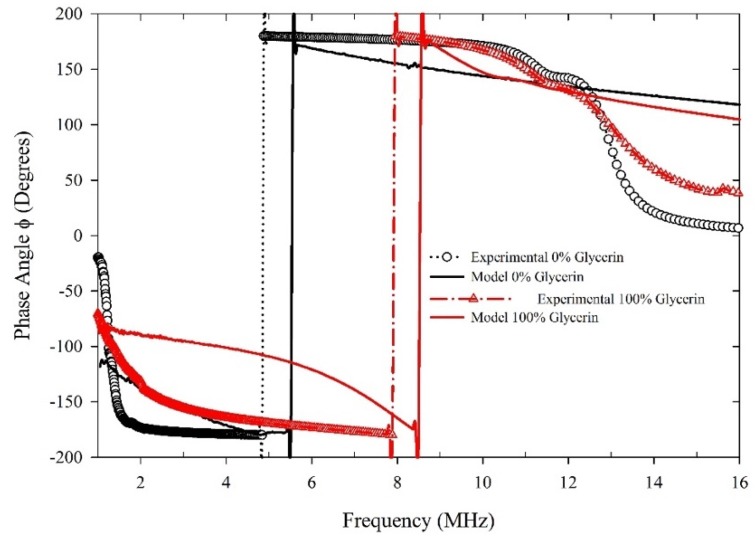
Variation of phase shift with frequency for both experimental and simulated at different concentration of glycerin and water.

Using ANSYS, numerical simulations, and analysis is performed. This analysis used a two-way FSI to find the phase shift actuator/sensor PZT pair under the excitation of the applied voltage. Figure 8 illustrates the phase shift change for the simulated and experimental of both distilled water and glycerin, respectively. Note that the trend of both results, numerical and experimental, follows the same pattern. The model shows that the phase shift change is at 5.5 MHz, and the experimental one is at 4.8 MHz for water. For glycerin, the values are 8.5 MHz and 7.8 MHz, respectively. The differences between the numerical and experimental results can be a result of several factors: liquid level in the device, slight manufacturing differences such as layer alignment within the probes many layers, and quality of adhesion, among others. The overall trend, however, seems to be quite similar. 

**Figure 9:**
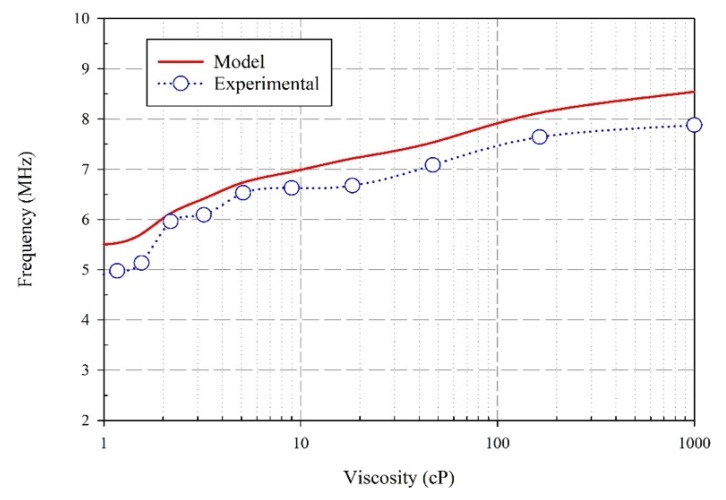
Variation of viscosity with resonant frequency for both experimental and modeled results.

All the cases tested experimentally are also modeled, and the results are shown in Figure 9. In spite of these differences, the trend is clear: higher viscosity results in a higher frequency shift. Future work includes exploring the limitations of the simulations of the model used here, such as the magnitude of the wave decays with distance.

## 6. Conclusions

A new method and probe are designed and tested to monitor viscosity changes in fluids. This probe consists of an actuator and a sensor, both made of a commercially available soft piezoelectric material (PZT). It is mainly designed around two constraints: size, to fit into a 3 mL vacutainer and cost: low-cost, and hence disposable. The operation of the device is based on a tuning fork principle as part of a mechanism for transmitting waves in a fluid medium. The actuator is powered with a sinusoidal signal such that it vibrates, generating an acoustic wave through a fluid. The sensor detects the produced wave in the liquid. The phase shift between the emitted and detected wave signal is recorded and plotted for different concentrations of glycerin in water. Numerical simulations through a finite element method (FEM) and the software ANSYS were performed. Acoustic modal and harmonic acoustic analysis results from the simulations predict the experimental results. The results from the modal analysis show that the first, second, and third modes of the probe are in the range of 683.9–2358 Hz for air, 499.6–1080.2 Hz for water, and 468.5–625.4 Hz for glycerin. From the harmonic acoustic analysis, the results show that the ultrasonic frequency for phase shift effects ranges from 6 to 9 MHz. These results demonstrate that the natural frequencies of the probe are in the kHz region, and the phase shift frequencies are predicted to be in the high-frequency range.

These particular results emphasize that the results obtained seemed to be directly linked to the (electric) acoustic impedance of the probe in a fluid as observed in the impedance measurements of the actuator and sensor. Results show a trend: a phase shift increases with the viscosity of the liquid. The results of this work also show that wall interference is not a contributing factor because the distance between the sensor and actuator is small. The main contribution of this device to the field of viscosity monitoring is the use of techniques used in structural health monitoring. The developed method also has many advantages over other devices such as size, portability, low cost, require minimal samples of liquid to operate, and it is disposable. All these characteristics enable multiple uses for such a method, such as inline monitoring and point-of-care devices. For example, it can be used in medical applications such as monitoring a person in an ambulance who suffers from a chronic disease. The approach and built probe are the initial steps for the development of a robust system. Accuracy and repeatability are yet not assessed since the manufacturing method is not commercially available. The device has not been tested with non-Newtonian or moving fluids, and those are research areas which need to be expanded numerically and experimentally. The method also may have other applications, such as acoustic pressure sensors or density monitoring.

## Figures and Tables

**Table 1:** Comparison of our probe with the existing type of devices.

Method	Viscometer Type	Typical Measurable Range (cP)	Price	Disposable
Displacement (1st type)	Capillary *	0.2–200	$$$	No
	Rotating *	0.3–320 M	$$$$$	No
Vibration/mass (2nd type)	Vibration *	0.3–100 k	$$$$	No
	MEMS **	variable	variable	No
Acoustic (3rd type)	Wave reflection ***	1.005–1400	variable	No
	Non-destructive	1.005–1600	$	Yes

* More detailed information available, Reference [8] (pp 819–820)

** More detailed information available, References [10,26]

*** More detailed information available, Reference [1].

**Table 2:** Mechanical properties for the viscosity probe [35].

Material	Piece #	Dimensions (mm)	Density (kg/m^3^)	Modulus of Elasticity (N/m^2^)
Hollow brass	1	5 × 5 × 70	8500	96 × 10^9^
Copper	2	6 × 0.1 × 25	8900	110 × 10^9^
PZT 5A	2	5 × 0.1 × 23	7550	

**Table 3:** Statistical analysis of the regression.

*R*	*R^2^*	Adjusted *R^2^*	Standard Error of Estimate	
0.9987	0.9975	0.9969	22.19	
	Coefficient	Std. Error	t	P
*a*	1341.292	342,891,640	3.91 × 10^−6^	1.00
*b*	0.115	118,380	9.73 × 10^−7^	1.00
*f_0_*	7.365	213,305	3.687 × 10^−5^	1.00
Analysis of Variance				
		DF	SS	MS
Regression		3	1,812,696	604,232
Residual		8	3940	492

**Table 4:** Natural frequency for the probe of different fluids in (Hz).

Fluid	Density (kg/m^3^)	Speed of Sound (m/s)	Mode 1	Mode 2	Mode 3
Air	1.2	343	683.86	684.24	2358
Water	1000	1484	499.88	533.88	1080.2
Glycerin	1260	1920	468.53	501.72	625.45
